# Molecular Characterisation and Descriptive Analysis of Carbapenemase-Producing Enterobacterales: Striking Epidemiological Changes

**DOI:** 10.3390/antibiotics15070713

**Published:** 2026-07-22

**Authors:** Estíbaliz Ugalde Zárraga, Matxalen Vidal-García, Mikel Urrutikoetxea-Gutiérrez, Elena Eraso, Itziar Angulo López, José Luis Díaz de Tuesta del Arco

**Affiliations:** 1 Clinical Microbiology Service, Basurto University Hospital, 48013 Bilbao, Spain; 2 Biobizkaia Health Research Institute, 48903 Barakaldo, Spain; 3 Department of Immunology, Microbiology and Parasitology, Faculty of Medicine and Nursing, University of the Basque Country (EHU), 48940 Leioa, Spain

**Keywords:** carbapenemase-producing Enterobacterales, metallo-β-lactamases, dual carbapenemase producing Enterobacterales, sequence types, mobile genetic elements

## Abstract

**Background/Objectives**: *Klebsiella pneumoniae* complex, *Escherichia coli*, and *Enterobacter cloacae* complex are considered the most prevalent carbapenemase-producing (CP) Enterobacterales in Spain. However, temporal changes in carbapenemase distribution, clonal diversity, and the mobile genetic elements (MGEs) involved in their dissemination have not been systematically characterised in our region. To address this knowledge gap, this study investigated the epidemiology of these organisms over a seven-year period. **Methods**: CP Enterobacterales isolates recovered between 2019 and 2025 from rectal screening swabs and clinical specimens (blood, urine, respiratory, wound, and sterile fluid samples) were included. All 297 isolates underwent phenotypic characterisation. Carbapenemase detection and sequence type (ST) assignment were performed for the 282 isolates available for molecular analysis. Following the gradual implementation of long-read whole-genome sequencing (WGS) beginning in 2022, MGEs associated with carbapenemase dissemination were characterised, and selected high-risk clones were analysed by SNP-based phylogenetic analysis. **Results**: Fifty isolates were recovered from ICU admission rectal screening, and 247 from clinical samples. Although the proportion of CP Enterobacterales among Enterobacterales isolates remained stable throughout the study period, significant changes were observed between 2019 and 2022 and between 2023 and 2025 in carbapenemase types, with a statistically significant increase in metallo-β-lactamases (MBLs) (*p* < 0.001), as well as in species distribution. Furthermore, predominant plasmid replicons associated with the dissemination of MBL genes were characterised. A *K. pneumoniae* ST147 clone co-harbouring *bla*_OXA-48_ and *bla*_NDM-1_ emerged and expanded significantly during the study period (*p* < 0.05). In addition, an ST225 NDM-1 clone was identified. SNP-based phylogenetic analysis revealed distinct genetic clusters within both lineages. **Conclusions**: Temporal shifts in the epidemiology of CP Enterobacterales, including the emergence and expansion of high-risk clones, were observed. High-resolution genomic analyses provided additional insight into the genetic relationships among circulating lineages.

## 1. Introduction

Carbapenemase production in Enterobacterales is a serious public health threat. For this reason, the World Health Organization included them in the critical group of the Bacterial Priority Pathogens List in 2017 and maintained them in the latest update in 2024 [1]. *K. pneumoniae* complex, *E. coli* and *E. cloacae* complex are among the most frequently reported carbapenemase-producing (CP) Enterobacterales in Spain [2], and their emergence and uncontrolled dissemination are of great concern. The epidemiology of carbapenemases varies markedly across geographical regions and healthcare settings, with important differences in the distribution of carbapenemase types reported even within Europe. In particular, while KPC enzymes remain predominant in some countries [3], an increasing prevalence of NDM-1 has been reported in others [4].

In our setting, a shift in carbapenemase epidemiology has been observed, including the emergence of MBL-producing Enterobacterales. These regional differences highlight the importance of understanding local epidemiological data for the effective control of hospital-acquired infections and the protection of public health.

The aim of this study is to describe the epidemiological scenario and perform genomic characterisation of CP-*Klebsiella pneumoniae* complex, CP-*Escherichia coli* and CP-*Enterobacter cloacae* complex isolates in a healthcare area serving approximately 360,000 inhabitants in northern Spain. Specifically, we analysed temporal trends in CP Enterobacterales isolation, the distribution of circulating clonal groups, and the dissemination of carbapenemase-carrying plasmids.

## 2. Results

### 2.1. Overall Epidemiology

From January 2019 to December 2025, 297 CP Enterobacterales isolates were identified among 64,687 isolates of *K. pneumoniae* complex, *E. coli*, and *E. cloacae* complex, corresponding to an overall proportion of 0.46% (297/64,687). No significant temporal variation was observed.

Fifty isolates (50/297; 16.8%) were obtained from rectal screening at intensive care unit admission, whereas 247 isolates (247/297; 83.2%) were obtained from clinical samples (blood, urine, respiratory, wound, and sterile fluid samples).

Of the 297 isolates, 207 (69.7%) were *K. pneumoniae* complex, 51 (17.2%) *E. coli*, and 39 (13.1%) *E. cloacae* complex. The yearly distribution of CP isolates showed mean proportions of 1.9% (207/10,817) for *K. pneumoniae* complex, 0.1% (51/50,770) for *E. coli* and 1.3% (39/3100) for *E. cloacae* complex.

For comparative analyses, the study period was divided into two intervals (2019–2022, 152 isolates; 2023–2025, 145 isolates), using 2022 as the cutoff year based on the detection of a statistically significant change in epidemiological trends.

### 2.2. Temporal Evolution of Carbapenemases

**Figure 1:**
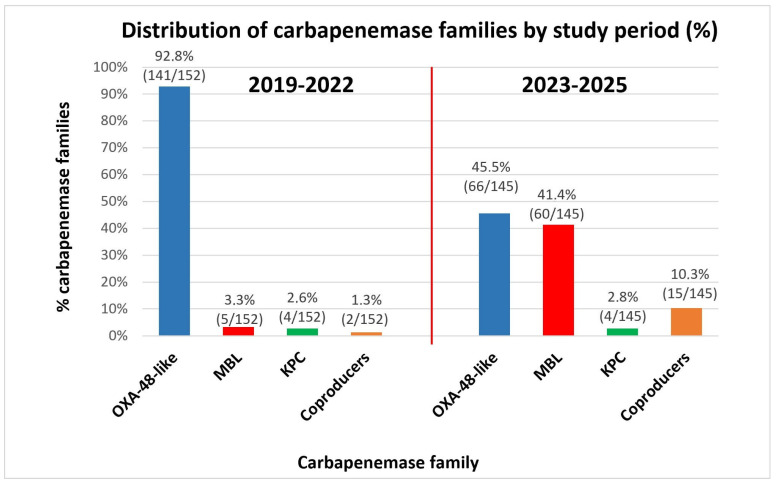
Distribution of carbapenemase families among carbapenemase-producing Enterobacterales during the two study periods (2019–2022 and 2023–2025).

A marked shift in carbapenemase epidemiology was observed between the two study periods (Figure 1). During 2019–2022, OXA-48-like enzymes predominated, accounting for 92.8% of all CP isolates (141/152; 95% CI, 87.5–95.9), whereas metallo-β-lactamases (MBLs) represented only 3.3% (5/152; 95% CI, 1.4–7.5). In contrast, during 2023–2025, the proportion of OXA-48-like enzymes declined significantly to 45.5% (66/145; 95% CI, 37.6–53.6), while MBL-producing isolates increased to 41.4% (60/145; 95% CI, 33.7–49.5) (Fisher’s exact test, *p* < 0.001).

A parallel increase was observed in dual carbapenemase-producing Enterobacterales (DCEB), whose prevalence rose from 1.3% (2/152; 95% CI, 0.4–4.7) during the first period to 10.3% (15/145; 95% CI, 6.4–16.4) during the second (Fisher’s exact test, *p* < 0.001). In contrast, KPC-producing isolates remained uncommon throughout the study, with a mean prevalence of 2.7%.

Temporal trends were evaluated using binomial generalised linear models (GLMs). These analyses confirmed significant year-on-year increases in both MBL-producing isolates (OR 1.52 per year, *p* < 0.001) and dual carbapenemase-producing Enterobacterales (DCEB) (OR 1.55 per year, *p* = 0.003).

**Figure 2:**
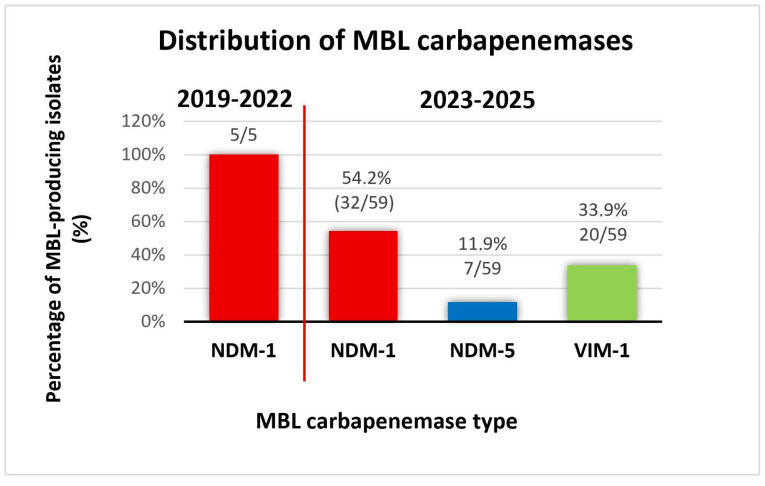
Distribution of MBL types during the study period.

Among the 282 isolates available for molecular characterisation (195 *K. pneumoniae* complex, 48 *E. coli*, and 39 *E. cloacae* complex), carbapenemase subtype diversity increased over time, particularly among MBLs. During 2019–2022, NDM-1 was the only MBL detected (5/5 isolates). However, during 2023–2025, VIM-1 and NDM-5 emerged, resulting in a more heterogeneous MBL population. Overall, NDM-1 remained the predominant MBL subtype, accounting for 54.2% (32/59) of MBL-producing isolates, followed by VIM-1 (20/59; 33.9%) and NDM-5 (7/59; 11.9%) (Figure 2).

### 2.3. Carbapenemase Distribution and Clonal Diversity by Species

#### 2.3.1. *K. pneumoniae* Complex

**Figure 3:**
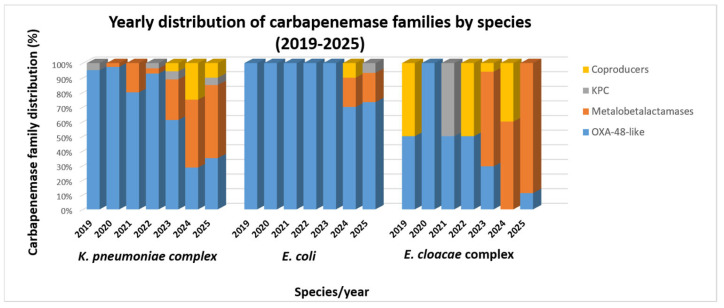
Yearly distribution of carbapenemase families among carbapenemase- producing *K. pneumoniae* complex, *E. coli*, and *E. cloacae* complex isolates between 2019 and 2025.

The *K. pneumoniae* complex was the predominant CP species throughout the study period, accounting for 207 of the 297 isolates (69.7%), including 123 isolates recovered during the first study period (2019–2022) and 84 during the second (2023–2025). A pronounced shift in carbapenemase epidemiology was observed over time (Figure 3). OXA-48-like enzymes predominated until 2022, whereas MBL-producing isolates progressively emerged from 2023 onwards. In 2023, MBLs accounted for 10 of 36 isolates (27.8%), and by 2024 they had become the most frequent carbapenemase family, exceeding OXA-48-like enzymes (13/28; 46.4% vs. 8/28; 28.6%). This trend continued in 2025, when MBLs represented 50.0% (10/20) of isolates, compared with 35% (7/20) producing OXA-48-like enzymes (Figure 3).

Within the OXA-48-like family, OXA-48 remained the predominant subtype throughout the study period, representing 99.0% (105/106) and 97.1% (33/34) of OXA-48-like isolates during the first and second study periods, respectively. Among MBL-producing isolates, NDM-1 remained the predominant subtype, although its relative frequency declined as additional MBL variants emerged. Whereas NDM-1 accounted for all five MBL-producing isolates detected during 2019–2022, it represented 66.7% (22/33) during 2023–2025, followed by VIM-1 (8/33; 24.3%) and NDM-5 (3/33; 9.1%).

**Figure 4:**
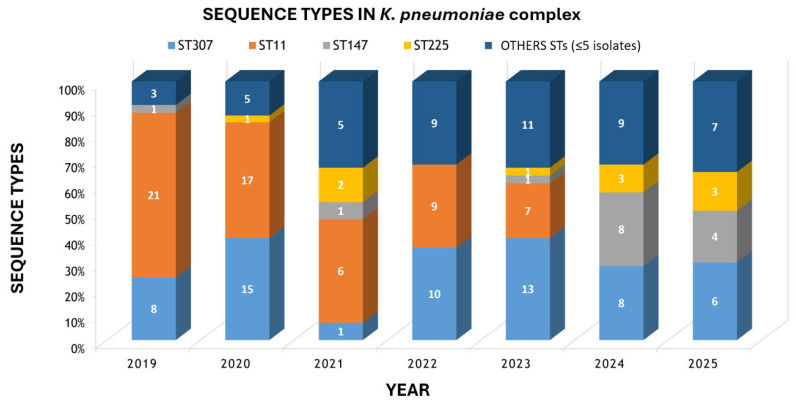
Annual distribution of the major sequence types (STs) among carbapenemase-producing *K. pneumoniae* complex isolates during the study period (2019–2025).

Thirty-nine sequence types (STs) were identified among the 195 genotyped *K. pneumoniae* complex isolates, indicating moderate genetic diversity. However, four lineages predominated: ST307 (61/195; 31.3%), ST11 (60/195; 30.8%), ST147 (15/195; 7.7%) and ST225 (10/195; 5.1%). Together, these four STs accounted for 74.9% of all sequenced isolates, whereas the remaining 35 STs were each represented by fewer than five isolates (Figure 4).

Comparison between the two study periods revealed a marked shift in the clonal population. During 2019–2022, ST307 and ST11 dominated the local epidemiology. In contrast, from 2024 onwards, ST11 disappeared, while ST307 persisted and was accompanied by the emergence of ST147, ST225 and several less frequent lineages (Figure 4). Notably, ST147 increased significantly over time (OR 1.93 per year, *p* < 0.001), highlighting the rapid expansion of this lineage since 2023.

The association between STs and carbapenemase genes also evolved over time (Table 1). ST11 remained consistently associated with *bla*_OXA-48_ throughout the study period. In contrast, ST307, which had been exclusively associated with *bla*_OXA-48_ until 2021, acquired a broader range of carbapenemase genes from 2022, reflecting the progressive emergence of MBLs within this successful lineage. Among the major STs, *bla*_NDM-1_ was the most frequently detected MBL gene (Table 1).

The emergence of dual CP-*K. pneumoniae* was first detected in 2023. The index isolate corresponded to an ST147 strain co-harbouring *bla*_OXA-48_ and *bla*_NDM-1_, recovered from a patient transferred to a chronic care hospital in our region ten days after discharge from a hospital in Ukraine. During 2024 and 2025, eight additional isolates carrying the same carbapenemase combination were identified. Five of these patients had been admitted to the same geriatric hospital several months after the Ukrainian patient, suggesting local dissemination of the clone.

Subsequently, two closely related variants of this lineage were also detected: two isolates carrying only *bla*_OXA-48_ following loss of *bla*_NDM-1_, and one isolate carrying only *bla*_NDM-1_ after loss of *bla*_OXA-48_.

Core genome MLST (cgMLST) analysis revealed that the nine dual-carbapenemase-producing isolates and the two related variants all belonged to cgST41315, supporting their close genetic relatedness.

**Figure 5:**
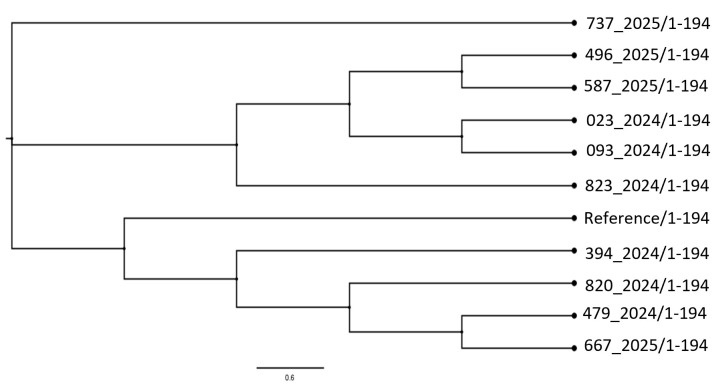
SNP-based phylogenetic tree of ST147 *Klebsiella pneumoniae* isolates. Two clades were identified. Clade I comprised isolates 023_2024, 093_2024, 587_2025, 496_2025, 737_2025 and 823_2024, whereas Clade II included isolates 394_2024, 667_2025, 820_2024, 479_2024 and the reference strain. In isolate identifiers, the second number corresponds to the year of isolation.

Single nucleotide polymorphism (SNP)-based phylogenetic analysis further resolved the ST147 isolates into two major clades, with pairwise SNP distances ranging from 10 to 99 SNPs. One clade comprised closely related isolates with pairwise distances of 10–30 SNPs, whereas the remaining isolates showed greater genetic divergence, with pairwise distances of up to 99 SNPs. Overall, these findings indicate that, although the isolates belonged to the same cgST, they comprised several closely related lineages rather than a single recently transmitted clone (Figure 5).

A second emerging lineage corresponded to ST225 carrying *bla*_NDM-1_. This clone was first detected in 2020 as two epidemiologically unrelated isolates. Between 2020 and 2025, seven additional isolates were identified, all recovered from residents of the same geriatric long-term care facility. All the isolates belonged to the same closest-defined cgST10757, supporting clonal dissemination within the institution.

**Figure 6:**
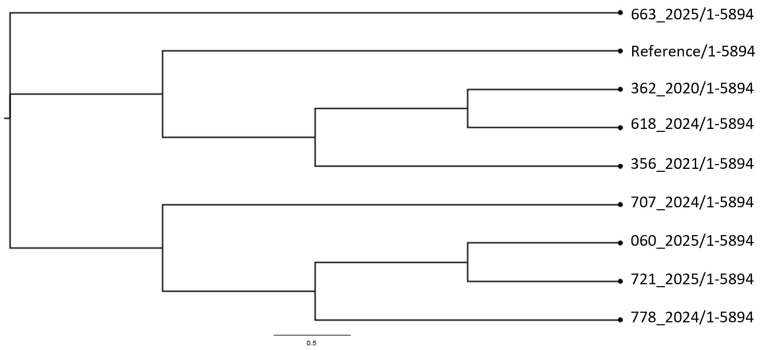
SNP-based phylogenetic tree of ST225 *Klebsiella pneumoniae* isolates. Two major clades were identified: Clade I included isolates 663_2025, 362_2020, 618_2024, 356_2021, and the earliest detected isolate (reference genome), whereas Clade II comprised isolates 707_2024, 060_2025, 721_2025, and 778_2024. The marked separation between clades indicates the circulation of two distinct genetic lineages. In isolate identifiers, the second number corresponds to the year of isolation.

Phylogenetic analysis based on whole-genome SNPs identified two well- defined clades separated by substantial genetic divergence, indicating the presence of two distinct genetic lineages. Clade I included four isolates and the earliest detected isolate, which served as the reference genome for the analysis, whereas Clade II comprised the remaining four isolates. Within each clade, isolates showed low genetic diversity, with pairwise distances ranging from 5 to 26 SNPs in Clade I and from 1 to 20 SNPs in Clade II, suggesting recent common ancestry. Overall, the genomic data support the presence of two potential transmission clusters rather than a single outbreak (Figure 6).

#### 2.3.2. *Escherichia coli*

A total of 51 CP-*E. coli* isolates were identified during the study period, with 21 isolates during 2019–2022 and 30 during 2023–2025. In contrast to the *K. pneumoniae* complex, OXA-48-like enzymes remained the predominant carbapenemase family throughout the study period (Figure 3). MBL-producing *E. coli* isolates were not detected until 2024, when the first NDM-1- and VIM-1-producing isolates emerged. Overall, MBLs remained uncommon, with only four isolates identified during the study period, comprising two NDM-1 producers and two VIM-1 producers. Likewise, dual carbapenemase production was rare, with a single OXA-48/NDM-5-producing isolate detected in 2024.

Among OXA-48-like enzymes, OXA-244 represented an important subtype in *E. coli*, accounting for 25.6% (11/43) of OXA-48 producers. Its prevalence increased significantly during the second study period, with seven of the eleven OXA-244-producing isolates (63.6%) detected in 2025 alone (Fisher’s exact test, *p* = 0.001).

A total of 28 STs were identified, confirming the highly diverse population structure of CP-*E. coli*. The internationally disseminated high-risk clone ST131, together with ST1193 and ST38, was the most frequently identified lineage; however, each represented only 8.3% (4/48). All remaining STs were represented by three or fewer isolates.

Among OXA-244-producing isolates, ST38 was the most frequently identified lineage, with all ST38 isolates carrying both *bla*_OXA-244_ and *bla*_CTX-M-27_. The remaining OXA-244-producing isolates belonged to several unrelated STs (ST58, ST162, ST501, ST636, ST648 and ST1722), further supporting the polyclonal dissemination of this carbapenemase.

Analysis of the genetic context showed that, in all eight sequenced OXA-244-producing *E. coli* isolates, *bla*_OXA-244_ was integrated into the chromosome as part of a composite transposon. The transposon was flanked either by IS1 family insertion sequences on both sides or by a combination of IS1 family and IS10A elements.

#### 2.3.3. *Enterobacter cloacae* Complex

Thirty-nine CP-*E. cloacae* complex isolates were recovered during the study period: eight during 2019–2022 and 31 during 2023–2025. Whole-genome sequencing (WGS) was performed for 17 isolates following the implementation of WGS at our institution, identifying 12 *Enterobacter hormaechei*, three *Enterobacter roggenkampii,* and two *Enterobacter kobei*.

Among the three species analysed, the *E. cloacae* complex exhibited the most pronounced temporal shift in carbapenemase epidemiology (Figure 3). During the first study period, isolates were almost exclusively associated with OXA-48-like enzymes, with only sporadic detection of KPC-producing isolates. From 2023 onwards, however, MBLs became the predominant carbapenemase family, accounting for 65% of isolates in 2023 and consistently exceeding OXA-48-like enzymes thereafter.

Within the OXA-48-like family, OXA-48 was the only subtype detected. In contrast, the MBL population became heterogeneous during the second study period. Among the 22 MBL-producing isolates identified, VIM-1 was the predominant subtype (10/22; 45.5%), followed by NDM-1 (8/22; 36.4%) and NDM-5 (4/22; 18.2%).

The *E. cloacae* complex also displayed a highly polyclonal population structure, with 17 different STs identified. Nevertheless, ST78 and ST1074 were the most prevalent lineages, accounting for 23.1% (9/39) and 10.3% (4/39) of isolates, respectively.

Among ST78 isolates, 77.8% (7/9) produced MBLs, including VIM-1 in 55.6% (5/9) and NDM-1 in 22.2% (2/9), while the remaining strains produced OXA-48. ST1074 was initially associated with OXA-48 in a single *E. roggenkampii* isolate recovered from the urine of a catheterized patient. In subsequent years, three additional ST1074 *E. roggenkampii* isolates from rectal carriers were identified, all of which coproduced OXA-48 and NDM-1.

By that time, only one DCEB had been identified (in 2019), corresponding to an ST93 *E. hormaechei* strain producing KPC-2 and VIM-1.

### 2.4. Genetic Location of MBL Genes

The genetic environment of the three major MBL genes (*bla*_NDM-1_, *bla*_NDM-5_, and *bla*_VIM-1_) was investigated to determine the mechanisms underlying their dissemination.

#### 2.4.1. *bla*_NDM-1_

*bla*_NDM-1_ gene was identified in three main genetic contexts, two of which were associated with specific *K. pneumoniae* clones, while the third was shared across multiple Enterobacterales species.

**Figure 7:**
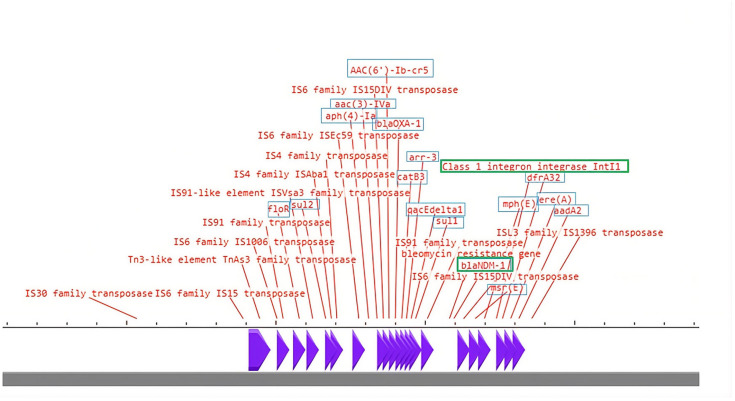
Genetic context of the chromosomal *bla*_NDM-1_ gene in ST225 *K. pneumoniae*. Green highlights the carbapenemase gene and its associated MGE; blue highlights the remaining resistance genes, and purple arrows indicate MGEs.

**Figure 8:**
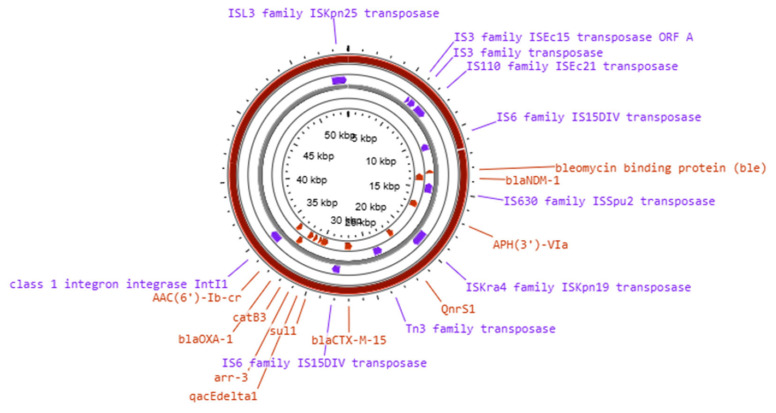
BLASTn comparison of the IncFIB(pQil) plasmid isolated at our institution (grey ring) with the NCBI reference plasmid OQ723099.1 (Spain; red ring), which is of Ukrainian origin. Purple: MGEs including integrons, transposons, and insertion sequences; red: antimicrobial resistance genes.

(a) In the nine sequenced ST225/NDM-1 *K. pneumoniae* isolates described above, *bla*_NDM-1_ was located on the chromosome within a class 1 integron flanked on both sides by the insertion sequence IS15DIV, a member of the IS6 family, forming a composite transposon. The integron also contained multiple additional resistance genes, including the bleomycin resistance gene (*ble*), *sul1*, *qacEΔ1*, *arr-3*, *catB3*, *bla*_OXA-1_, and *aac(6′)-Ib-cr*, among others. In addition, the chromosome also carried *bla*_CTX-M-65_ (Figure 7).

(b) A second genetic context was identified in the ST147 clone (cgST41315), in which *bla*_NDM-1_ was located on an approximately 52-kb IncFIB(pQil) plasmid. This plasmid showed 100% coverage and 100% identity to OQ723099.1 (Figure 8).

(c) The third and most widely distributed genetic context involved IncF plasmids circulating across different species.

**Figure 9:**
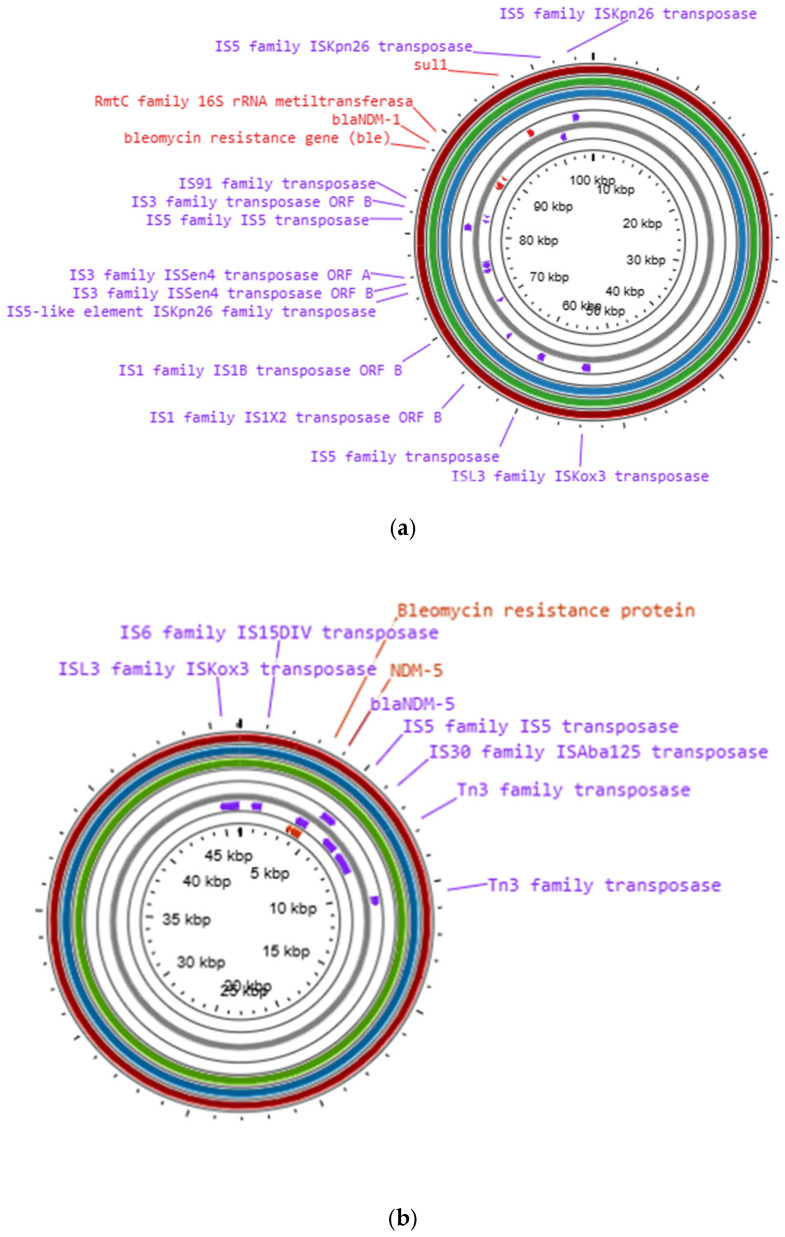

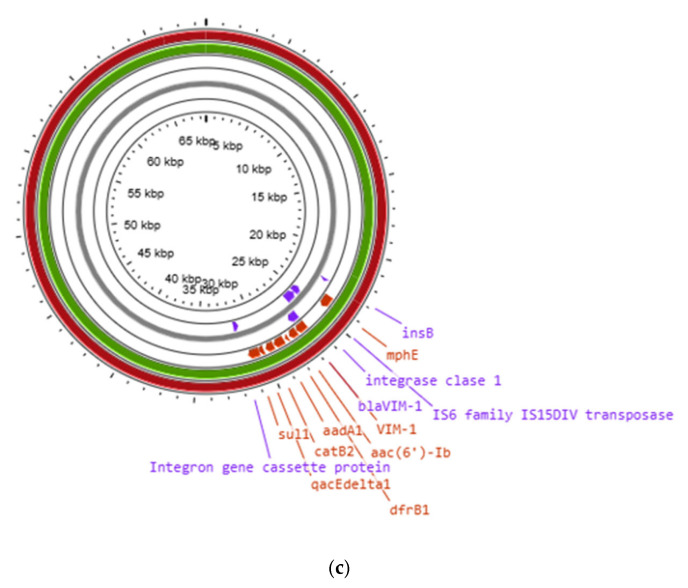
Reconstruction of plasmid with the most frequent replicon: (**a**) IncFII(Yp), carrying *bla*_NDM-1_ in *E. coli* (grey ring), *K. pneumoniae* (blue ring), and *E. kobei* (green ring) at our institution and BLASTn comparison with the NCBI reference plasmid KC887916.2 (red ring). (**b**) IncX3 replicon carrying *bla*_NDM-5_ in *K. pneumoniae* (grey ring), *E. coli* (green ring), and *E. hormaechei* (blue ring) at our institution and BLASTn comparison with the NCBI reference plasmid CP050157.1 (red ring). (**c**) IncL replicon harbouring *bla*_VIM-1_ in *K. pneumoniae* (grey ring), *E. hormaechei* (green ring) at our institution and BLASTn comparison with the NCBI reference plasmid LR991404.1 (red ring). Purple: MGEs including integrons, transposons, and insertion sequences; red: antimicrobial resistance genes.

Seventeen of the remaining 22 sequenced NDM-1-producing strains (77.3%), including three OXA-48 + NDM-1 *E. roggenkampii* isolates, harboured *bla*_NDM-1_ on IncF type plasmids. Of these, 14 (82.3%)—comprising 9 *K. pneumoniae*, 1 *E. coli*, 3 *E. roggenkampii* and 1 *E. kobei*—carried a single-replicon IncFII(Yp) plasmid (~105Kb), while 3 isolates (17.6%)—1 *K. pneumoniae* and 2 *E. hormaechei —*carried an IncFII(Yp)/IncFIB(pB171) multireplicon plasmid of the same size. These plasmids harboured a limited number of resistance genes, *bla*_NDM-1_, *sul1*, *ble,* and a *rmtC* family 16S rRNA methyltransferase gene, and showed 100% coverage and 100% identity to KC887916.2 (Figure 9a).

#### 2.4.2. *bla*_NDM-5_

In contrast, *bla*_NDM-5_ showed a distinct genetic context.

A ~46 kb single-replicon IncX3 plasmid carrying *bla*_NDM-5_ was identified in 8 of the 9 sequenced NDM-5-producing strains (2 *K. pneumoniae*, 1 *Klebsiella variicola,* 1 *Klebsiella quasipneumoniae*, 3 *E. hormaechei*, and 1 *E. coli*). These plasmids carried only two resistance genes, *bla*_NDM-5_ and *ble*. They matched CP050157.1 with 93% coverage and 99.97% identity (Figure 9b).

#### 2.4.3. *bla*_VIM-1_

Among the 21 VIM-1-producing isolates identified during the study period, including one *E. hormaechei* VIM-1/KPC-2 coproducer, 14 were sequenced. IncL plasmids were detected in 71.4% (10/14) of the sequenced isolates. Eight of these plasmids, recovered from five *K. pneumoniae* and three *E. hormaechei* isolates, were approximately 70 kb in size. They matched LR991404.1 with 100% coverage and 100% identity (Figure 9c). The remaining two IncL plasmids (1 *E. hormaechei* and 1 *E. coli*) were slightly larger, but in all cases, *bla*_VIM-1_ was located within a class 1 integron.

Overall, pairwise BLASTn comparisons of representative plasmids recovered from different bacterial species showed nucleotide identities ranging from 99.93% to 99.99%, with query coverage ranging from 93% to 100%, supporting the presence of highly similar plasmids across species.

## 3. Discussion

In this study, the epidemiology of CP Enterobacterales was analysed over a seven-year period. The overall proportion of CP isolates was 0.46%, consistent with previous national data [5]. However, unlike studies from other settings that have reported increasing trends [5,6], this proportion remained stable throughout the study period. Despite this overall stability, two distinct epidemiological phases were identified, marked by changes in species distribution, with the *E. cloacae* complex and *E. coli* becoming increasingly prevalent, together with shifts in the distribution of carbapenemase types. In agreement with recent studies from across Europe, a changing epidemiological landscape was observed, characterised by the statistically significant predominance of MBL genes [7], particularly NDM variants [8], and the increasing detection of isolates harbouring two carbapenemase genes, such as *bla*_OXA-48_ and *bla*_NDM_ [9]. The introduction of an ST147 *K. pneumoniae* clone co-producing OXA-48 and NDM-1 is particularly noteworthy. This high-risk clone (HRC) was first detected in our area in August 2023 and has recently been reported elsewhere in Spain [10]. In the context of population displacement associated with the war in Ukraine, these findings suggest that such events may facilitate the introduction of novel clones into previously unaffected healthcare settings.

In March 2022, the European Centre for Disease Prevention and Control (ECDC) recommended pre-emptive isolation and screening for multidrug-resistant bacteria, particularly carbapenem-resistant Enterobacterales, in patients transferred from Ukrainian hospitals or with a history of hospitalisation in Ukraine within the previous 12 months [11]. This recommendation is supported by the high rates of antimicrobial resistance reported in Ukraine, particularly among Gram-negative bacteria, as well as by surveillance data from several European countries [12,13], which documented the introduction of NDM-producing and OXA-48/NDM co-producing Enterobacterales associated with these patients.

Another notable finding was the recent increase in OXA-244-producing *E. coli*, predominantly observed during the final year of the study. Although OXA-244 was first described in Spain in a *K. pneumoniae* isolate in 2013 [14], a marked expansion of chromosomally encoded OXA-244 among *E. coli* isolates has been documented across several European countries [15,16].

Regarding the *E. cloacae* complex, the rise in carbapenemase production over recent years has not been consistently documented in the literature, except in specific settings such as Japan [17] and the United States [18]. In our cohort, however, a pronounced increase became evident in 2023. VIM-1 was the predominant carbapenemase among these isolates, as reported for this species. Notably, Spain was among the first Western European countries to document VIM-producing *Enterobacter* spp. [19].

Among the identified STs, ST307 and ST147 were the most prevalent *K. pneumoniae* clones in our setting. Both are recognised as HRCs because of their ability to disseminate globally, and they have been associated with multiple carbapenemases, including OXA-48, NDM-1, VIM-1, and their combinations [20,21]. Interestingly, the emergence of ST147 coincided with the decline of ST11, which had been frequent in our region along with ST307 until 2023. Although this finding suggests a shift in the distribution of dominant clones, a causal replacement cannot be inferred from our data. In addition, the detection of ST225 deserves attention. All isolates produced NDM-1, with the gene located on the chromosome. To our knowledge, this ST225 lineage has not previously been described, although its distribution and route of introduction remain unclear.

The combination of cgMLST and SNP-based phylogenetic analysis provided a more refined characterisation of the circulating HRCs. While cgMLST grouped the isolates within the same clonal lineage, SNP analysis identified distinct clusters, suggesting the co-circulation of closely related lineages rather than a single outbreak. These findings highlight the added value of integrating high-resolution genomic analyses with epidemiological data to investigate transmission events.

In the case of *E. coli* and the *E. cloacae* complex, both species showed considerable genetic heterogeneity, consistent with previous reports. Concerning *E. coli*, the high-risk ST131, together with ST1193 (described as a sister clone) and ST38, predominated among the identified STs. All have been described as extraintestinal pathogenic *E. coli* [22]. Regarding ST38, there is growing concern about its impact across the three axes of the One Health concept (humans, animals, and environment), particularly given its role in the spread of antibiotic resistance [23]. Several outbreaks in Germany and Switzerland caused by this clone carrying *bla*_OXA-244_ and *bla*_CTX-M-27_ were described in 2020 [24]. In our study, three isolates were identified (one in 2019 and two in 2025); however, due to the limited number and temporal distribution of isolates, we cannot conclude that this represents an established clone in our setting.

In the *E. cloacae* complex, ST78—the predominant clone in our area—is a well-recognized high-risk lineage [25] and, in our collection, was exclusively linked to *E. hormaechei*. However, ST1074, the second most frequent ST, was confined to *E. roggenkampii* and was mainly associated with OXA-48+NDM-1 -producing isolates from carrier screening samples. To date, this ST has not been described in the literature. Nevertheless, according to data from the Spanish carbapenemase surveillance program, it remains restricted to *E. roggenkampii* and has been associated with multiple carbapenemases [26].

Overall, these findings suggest a strong association between specific lineages and carbapenemase types. However, the presence of shared MGEs and the high sequence similarity among representative plasmids recovered from different bacterial species suggest that closely related plasmids have contributed to the interspecies spread of carbapenemase genes.

IncF replicons, particularly IncFII(Yp) [27], were associated with the dissemination of *bla*_NDM-1_, whereas IncX3 [28] plasmids were associated with *bla*_NDM-5_ and IncL [29] plasmids with *bla*_VIM-1_. Together with the introduction of ST147 and ST225 *K. pneumoniae* clones, these plasmids may have played a key role in the dissemination of MBLs and could partly explain their increasing prevalence in our region.

This study has several limitations. Its single-region design may restrict the extrapolation of the results to other geographical settings or healthcare systems. In addition, combining screening and clinical isolates may have influenced the observed distribution of CP Enterobacterales and HRCs. A further limitation was the unavailability of genotypic data for 15 of 297 isolates (5.1%) due to the lack of cryopreservation. Nevertheless, the small proportion of missing isolates is unlikely to have affected the overall results. WGS was implemented only from 2022 onwards, resulting in incomplete genomic characterisation of isolates collected during the early years of the study. Future multicentre studies with integrated genomic and clinical data are warranted to validate and extend these findings.

## 4. Materials and Methods

### 4.1. Study Design

This is a descriptive observational study conducted at Basurto University Hospital between January 2019 and December 2025. This study was approved by the Ethics Committee of the Basurto University Hospital, Bilbao, Spain (approval no. 76.22 CEIHUB).

### 4.2. Inclusion and Exclusion Criteria

- Inclusion criteria
  1. Isolates obtained from intensive care unit admission screening (carriage) and clinical samples collected from an acute tertiary hospital, a chronic disease hospital, and outpatient services.
  2. Only the first isolate per patient during the study period was included.
- Exclusion criteria
  1. Subsequent isolates from the same patient were excluded unless they were recovered from blood, in which case the bloodstream isolate replaced the initial isolate.

### 4.3. Isolates Identification

Bacterial identification was performed by MALDI-TOF MS using the MALDI Biotyper system (Bruker Daltonics, Bremen, Germany). Identification of members of the *Enterobacter cloacae* complex was confirmed by WGS when available [19].

### 4.4. Antimicrobial Susceptibility Testing and Phenotypic Carbapenemase Resistance Identification

Antimicrobial susceptibility testing was performed using the automated MicroScan WalkAway (Beckman Coulter Diagnostics, Brea, CA, USA) system with NM63 panels. Carbapenemase production was screened using a meropenem MIC cutoff of >0.125 mg/L in piperacillin/tazobactam-resistant isolates, following the guidelines of the European Committee on Antimicrobial Susceptibility Testing (EUCAST) [30], as a sensitive screening threshold.

Phenotypic characterisation was carried out by immunochromatography (NG-Test CARBA 5, NG Biotech, Guipry-Messac, France).

### 4.5. Genotypic Characterisation

Genomic DNA was extracted either by the boiling method or with the MagLEAD 12gC automated nucleic acid extraction and purification platform (Precision System Science, Matsudo, Chiba, Japan) for sequencing, which routinely yielded DNA concentrations of 50–300 ng/μL.

**Figure 10:**
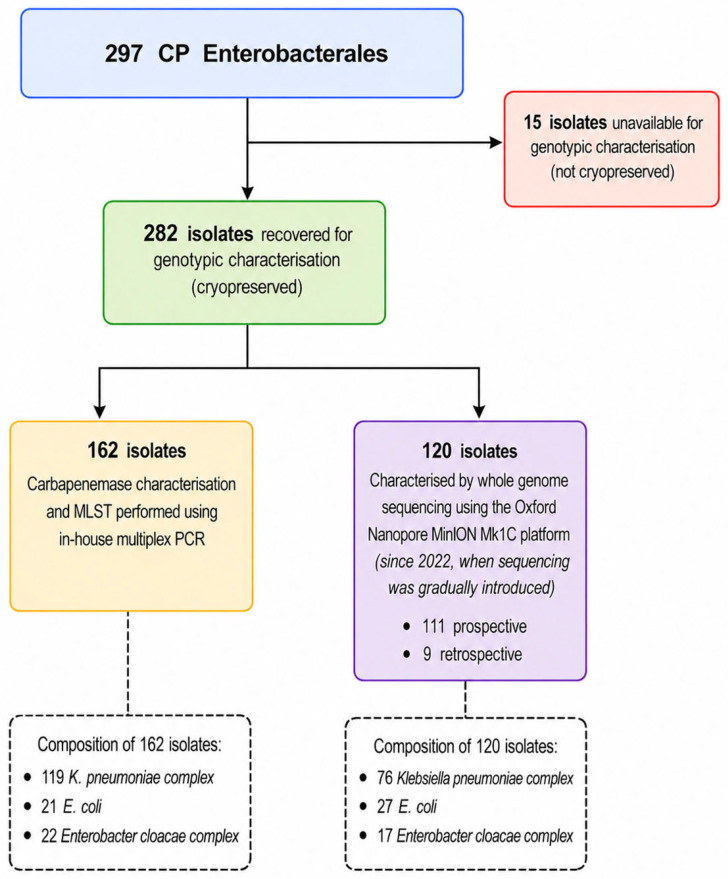
Genotypic characterisation workflow.

The workflow for genotypic characterisation is summarised in Figure 10.

Carbapenemase characterisation and multilocus sequence typing (MLST) were performed using in-house multiplex PCR and the Pasteur (*K. pneumoniae* complex), Achtman (*E. coli*), and *E. cloacae* complex MLST schemes, respectively.

WGS was performed using the Oxford Nanopore MinION Mk1C platform, based on long-read sequencing technology [31]. The samples were sequenced on R10 flow cells employing the Rapid Barcoding Kit 96 V14 chemistry (SQK-RBK114.96, Oxford Nanopore Technologies, Oxford, UK). Sequencing yielded reads with an N50 value of 30kb, achieving a sequencing depth of 35× and a genome coverage of 99%. The obtained reads were basecalled with Dorado v1.0.0 in superior accuracy mode, and the resulting FASTQ files were quality-checked using NanoPlot v1.x before de novo assembly with Flye v2.9.5 [32]. Antimicrobial resistance gene detection and MLST analysis for the *K. pneumoniae* complex were performed using the Pathogenwatch platform (https://pathogen.watch/, accessed on 15 December 2025). For the other species, ResFinder and MLST tools from the Center for Genomic Epidemiology (CGE; http://www.genomicepidemiology.org/, accessed on 25 December 2025) were used. cgMLST analysis was performed using MBioSEQ™ Ridom Typer (Bruker, Bremen, Germany) according to the *K. pneumoniae* cgMLST scheme (2358 target genes). SNP-based phylogenetic analysis was performed using CSI Phylogeny v1.4 [33], and phylogenetic trees were visualized with FigTree v1.4.4 (http://tree.bio.ed.ac.uk/software/figtree/, accessed on 10 July 2026).

Plasmid analysis was performed using PlasmidFinder and the Mobile Genetic Elements (MGE) database (CGE web servers, accessed on 20 January 2026). Plasmids were reconstructed with Proksee [34] after contig selection using SnapGene Viewer v7.2.1 (GSL Biotech), integrating Bakta annotation [35] and the Comprehensive Antibiotic Resistance Database (CARD) Resistance Gene Identifier (RGI). Sequence similarity and circular plasmid comparisons were performed using BLAST (https://blast.ncbi.nlm.nih.gov/Blast.cgi, accessed on 20 April 2026) and Proksee, respectively.

Representative plasmid sequences, one from each major plasmid type identified in this study, were deposited in GenBank under accession numbers PZ670939, PZ670940, PZ670941, and PZ670942.

### 4.6. Statistical Analyses

All proportions were calculated using the total number of non-duplicate isolates as the denominator (overall or by bacterial species, as appropriate). Proportions and their 95% confidence intervals (95% CI) were calculated using the Wilson score method. Differences in the distribution of carbapenemase families, carbapenemase subtypes, and sequence types between study periods were assessed using Fisher’s exact test in Jamovi (version 2.6.44). A *p* value < 0.05 was considered statistically significant.

Temporal trends in MBL-producing isolates, dual CP Enterobacterales and the proportion of ST147 among CP-*K. pneumoniae* complex isolates were assessed using generalised linear models (GLMs), with year as a continuous predictor. Analyses were performed using R software (version 4.4.0).

## 5. Conclusions

Monitoring isolates over time allows the detection of relevant epidemiological shifts. In our area, carbapenemase epidemiology has evolved from a pattern dominated by OXA-48-like enzymes towards a more complex scenario, characterised by a sustained increase in MBLs, particularly NDM-type enzymes, a broader distribution across Enterobacterales species, and an increase in carbapenemase co-production. These findings suggest, at least in part, the interspecies dissemination of resistance determinants mediated by MGEs, highlighting the role of plasmid-driven transmission of carbapenemase genes [36].

From a clinical perspective, carbapenemase co-producing isolates, particularly OXA-48 + NDM-1 producers—the most frequently reported carbapenemase combination [37]—represent a major epidemiological and therapeutic challenge, frequently limiting treatment options.

Finally, the emergence and expansion of HRC contributed to the dissemination of carbapenem-resistant Enterobacterales, while WGS, complemented by SNP-based phylogenetic analysis, represents a valuable tool for epidemiological surveillance, plasmid characterisation, and the investigation of transmission dynamics [38].

## Figures and Tables

**Table 1:** Evolution of ST/carbapenemase combinations in major STs in *K. pneumoniae*.

		2019	2020	2021	2022	2023	2024	2025
**ST307**	**OXA-48**	8	15	1	8	6	4	3
	**NDM-1**				1	3	2	3
	**NDM-5**					1		
	**VIM-1**					3	2	
	**KPC-3**				1			
**ST11**	**OXA-48**	21	17	6	9	7		
**ST147**	**OXA-48+NDM-1**					1	6	2
	**OXA-48**						1	2
	**NDM-1**			1			1	
	**KPC-2**	1						
**ST225**	**NDM-1**		1	2		1	3	3

Colour coding: Blue, OXA-48-like carbapenemases; orange, MBLs; yellow, coproduction of OXA-48 and NDM-1; grey, KPC variants. Blank cells indicate zero isolates. Molecular data correspond to genotyped isolates.

## Data Availability

The nucleotide sequences of the four representative plasmids have been deposited in GenBank under accession numbers PZ670939, PZ670940, PZ670941, and PZ670942.
